# Prevalence of Small Intestinal Bacterial Overgrowth among Chronic Pancreatitis Patients: A Case-Control Study

**DOI:** 10.1155/2016/7424831

**Published:** 2016-04-03

**Authors:** Amelie Therrien, Simon Bouchard, Sacha Sidani, Mickael Bouin

**Affiliations:** Laboratoire de Neurogastroentérologie et Motricité Digestive, Service de Gastroentérologie, Hôpital Saint-Luc, Centre de Recherche du Centre Hospitalier de l'Université de Montréal (CRCHUM), Montréal, QC, Canada H2X 3J4

## Abstract

*Background*. Patients with chronic pancreatitis (CP) exhibit numerous risk factors for the development of small intestinal bacterial overgrowth (SIBO).* Objective*. To determine the prevalence of SIBO in patients with CP.* Methods*. Prospective, single-centre case-control study conducted between January and September 2013. Inclusion criteria were age 18 to 75 years and clinical and radiological diagnosis of CP. Exclusion criteria included history of gastric, pancreatic, or intestinal surgery or significant clinical gastroparesis. SIBO was detected using a standard lactulose breath test (LBT). A healthy control group also underwent LBT.* Results*. Thirty-one patients and 40 controls were included. The patient group was significantly older (53.8 versus 38.7 years;* P* < 0.01). The proportion of positive LBTs was significantly higher in CP patients (38.7 versus 2.5%:* P* < 0.01). A trend toward a higher proportion of positive LBTs in women compared with men was observed (66.6 versus 27.3%;* P* = 0.056). The subgroups with positive and negative LBTs were comparable in demographic and clinical characteristics, use of opiates, pancreatic enzymes replacement therapy (PERT), and severity of symptoms.* Conclusion*. The prevalence of SIBO detected using LBT was high among patients with CP. There was no association between clinical features and the risk for SIBO.

## 1. Introduction

Small intestinal bacterial overgrowth (SIBO) is defined as the presence of >10^5^ bacteria/mL in the small bowel, most of which are enterobacteria from the colonic flora [1]. Such overgrowth can cause malabsorption and maldigestion, which subsequently leads to diarrhea, steatorrhea, bloating, chronic pain, and vitamin B_12_ deficiency [1]. The main risk factors for SIBO include conditions associated with stasis of intestinal content due to structural abnormalities or dysmotility [1–6] as well as conditions associated with achlorhydria [1, 2]. There is controversy regarding the possibility of an association between SIBO and the use of proton pump inhibitors [1, 2, 7]. Moreover, SIBO may represent both a cause and a consequence of slow intestinal transit [8, 9].

Chronic pancreatitis (CP) is a cause of abdominal pain, steatorrhea, and malabsorption and can lead to significant narcotic use [10]. Many factors are associated with both CP and SIBO, especially alcohol and narcotic use, intestinal dysmotility, and pancreatic enzyme deficiency [11]. It is hypothesized that pancreatic enzymes have antimicrobial properties and modify the intestinal chyme [12, 13] and that a deficiency leads to SIBO. Although previous studies have suggested a high incidence of SIBO in patients with CP [1, 14–17], small sample size and confounding factors, such as narcotic use and history of gastroduodenal surgery (with or without vagotomy), render a direct association between CP and SIBO difficult [13–17].

We hypothesized that there is a high prevalence of SIBO in patients with CP, which may contribute to the chronicity of diarrhea and bloating. The aim of the present study was to determine the prevalence of SIBO among patients with CP. Second, due to the similarities in the clinical presentation of these two diseases, we also sought to identify more specific clinical factors for SIBO among patients with CP.

## 2. Methods

A prospective case-control study was performed between January and September 2013 at the digestive motility laboratory of the Centre Hospitalier de l'Université de Montréal (Montreal, Quebec). The study protocol was approved by the Ethics Committee of the institution on January 8, 2013. Informed written consent was obtained from all patients.

### 2.1. Patients

Patients with CP who were followed up by a gastroenterologist or a hepatobiliary surgeon in the authors' tertiary-care centre were recruited.

### 2.2. Inclusion Criteria

Patients between 18 and 75 years of age with a confirmed diagnosis of CP were included in the present study. Diagnosis was documented with either an abdominal computed tomodensitometry (according to Cambridge criteria [18]), a magnetic resonance imaging (MRI), or an endoscopic ultrasound (Rosemont criteria [19]). Patients >75 years of age, without diagnostic imaging documentation, who had gastroparesis or severe diabetes or were unable to fast before the lactulose breath test (LBT) were excluded. Patients with a history of gastric, intestinal, or pancreatic surgery (except appendectomy and cholecystectomy) as well as patients who underwent antibiotic therapy in the past month were also excluded. Patients taking prokinetic agents, probiotics, or laxatives were asked to discontinue the medications at least seven days before the test.

### 2.3. Control Group

The control group consisted of 40 healthy subjects between 18 and 75 years of age who agreed to undergo an LBT. Exclusion criteria were current chronic disease, a history of intestinal resection, or having taken any medication in the two weeks preceding the test. Every healthy subject provided written consent.

### 2.4. LBT

The LBT is a standardized and validated test to determine the presence of SIBO and to calculate intestinal transit time [20]. Lactulose is a sugar that is not absorbed in the intestine and becomes fermented by colonic bacteria after ingestion. An early production of hydrogen in the first 90 min is considered to be the result of fermentation by small bowel bacteria [20]. Some patients have methanogenic bacterial flora and, thus, measurements of methane are also performed [20–22].

Participants were required to fast for at least 8 h before the breath test and had to follow a special diet the day before (FODMAP diet without fiber, nonfermentable carbohydrates, and lactose). Smoking within 2 h of the test was prohibited.

The participants were instructed to blow into a Quintron 12i (QuinTron Instrument Company, Inc., USA) bag after washing their mouth with a solution of chlorhexidine 0.12% to eliminate oral flora bacteria. The hydrogen, methane, and carbon dioxide concentration (in parts per million (ppm)) were analysed using a MicroLyser Plus (QuinTron Instrument Company, Inc., USA). Subjects with an initial hydrogen value >20 ppm were invited to repeat the test at a later date and asked again about their diet and smoking before the test. The remainder of the participants were then given 10 g of lactulose to swallow. Measurements of expired gas were taken every 15 min for the following 3 h.

Test results that were considered to be positive for SIBO included an increase in hydrogen concentration >20 ppm over the baseline value in the first 90 min, two consecutives peaks >13 ppm noted, one of which occurring before 90 min, and two tests with a baseline hydrogen value >20 ppm, if the patient was deemed reliable on having followed the diet before the test [23–25]. Every hydrogen value was corrected by the device according to the dead space (exhaled carbon dioxide measurement).

### 2.5. Data Collection

A chart review was performed to collect patient clinical data. A standardized questionnaire was completed by the patients the day of the breath test. This questionnaire explored patient demographics, medical and surgical history, medication use, and recent gastrointestinal symptoms and their severity. The symptom inquiry was based on the gastrointestinal symptom score used for functional dyspepsia [25]. Symptoms specific to CP were added [10]. A scale of 0 to 4 was used to grade symptom severity, with a score of 3 or 4 considered to be severe. Narcotic use was graded from occasional use during pain exacerbations to regular intake; use in the 24 h preceding the test was also documented. A score using the M-ANNHEIM severity index was calculated for each participant. The latter is one of the most recent scoring systems incorporating clinical parameters such as pain, severe complications, pancreatic exocrine, and endocrine insufficiency as well as pancreas morphology based on the Cambridge classification [18]. Finally, demographic data and body mass index of the healthy control subjects were collected on the day of the breath test.

### 2.6. Statistical Analysis

Considering an expected prevalence of SIBO of 2% to 5% in the control group and 35% to 50% in patients, it was estimated that 25 to 30 patients with CP were required to achieve significance and adequately test for a difference in the prevalence of SIBO between CP patients and the 40 healthy controls [1, 14, 15, 17]. A comparative analysis of the demographic and clinical data was performed between the two groups. The aim of this analysis was to determine whether certain clinical characteristics predispose to SIBO. Fisher's exact test was used for this statistical analysis. Finally, the *t*-test was used to compare the means of hydrogen concentration at every measurement time between the CP and control groups. It was also used for the comparison of the elapsed mean times before reaching a colonic peak, being defined as the first increase in hydrogen >20 ppm over the baseline, if happening after 90 min, or a second increase >20 ppm in the event of a first peak before 90 min.

## 3. Results

### 3.1. Participant Characteristics

Forty CP patients and 40 controls were evaluated. Nine patients with CP were excluded because of lack of cooperation during the protocol (*n* = 2) or due to unwillingness to repeat the test a second time after an initial breath test result >20 ppm (*n* = 7). Thus, 31 patients with CP were included in the study. Their clinical characteristics are summarized in Table 1. Compared with the control group, CP patients were significantly older (53.8 years versus 38.7 years; *P* < 0.01) and included fewer women (29.0% versus 87.5%; *P* < 0.01).

### 3.2. Clinical Characteristics of CP Patients

**Figure 1:**
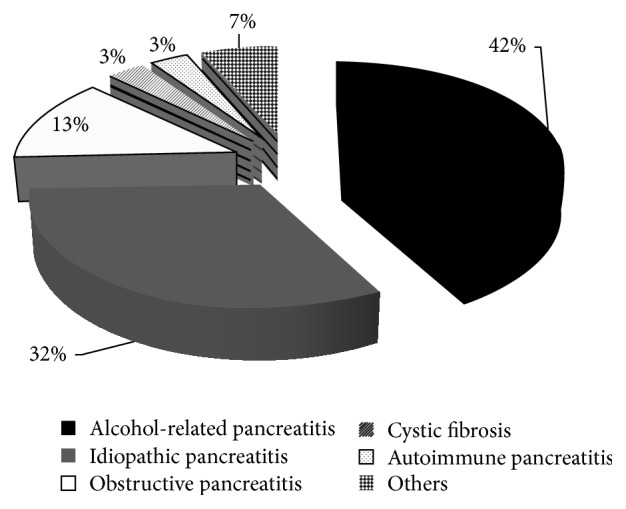
Etiology of chronic pancreatitis.

The different causes of pancreatitis in the CP group are presented in Figure 1. The etiology of CP was alcohol in 42% of the patients. The clinical manifestations and/or symptoms are presented in Table 2. The imaging modality used for diagnosis of CP was endoscopic ultrasound in 17 patients, abdominal computed tomography in 10, and abdominal magnetic resonance imaging in four. Regular medication taken by CP patients was as follows: 52.0% taking a proton pump inhibitor, 51.6% prescribed pancreatic enzyme replacement therapy, 22.6% taking narcotics, 12.9% using prokinetic agents, and 3.1% using probiotics. Based on the M-ANNHEIM severity index, clinical severity of the pancreatitis was considered to be minor for 14 patients, increased for 11, advanced for four, and marked for two [18].

### 3.3. LBTs

**Figure 2:**
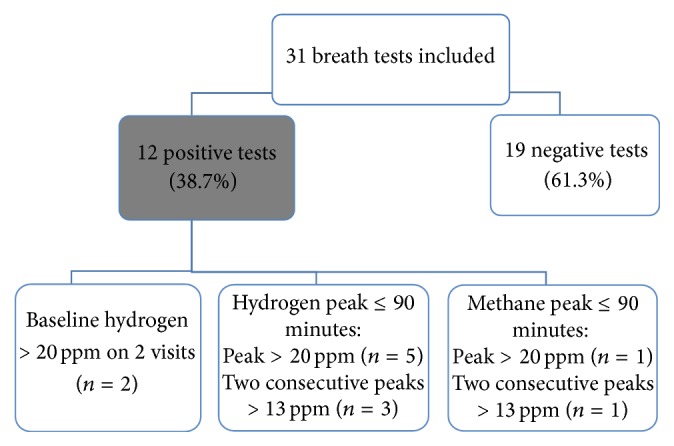
Breath test results of patients with chronic pancreatitis, ppm: parts per million.

The test was suggestive of bacterial overgrowth in 12 patients. The numbers of positive test results according to each positivity criterion with hydrogen and methane are presented in Figure 2. The LBT was well tolerated and the only adverse event was diarrhea during the test in one patient (LBT negative for SIBO).

**Figure 3:**
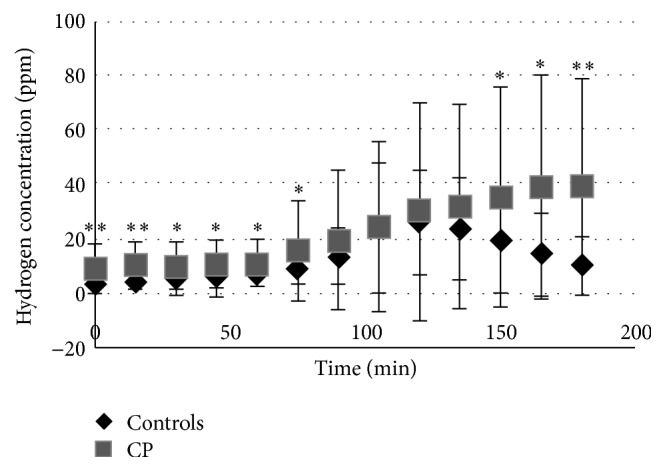
Mean ± SD values of exhaled hydrogen concentration (parts per million (ppm)) during lactulose breath test (LBT). Comparison of the mean curves and SD of exhaled hydrogen concentration of LBT between chronic pancreatitis (CP) patients and controls; ^*∗*^ *P* < 0.05; ^*∗∗*^ *P* < 0.0001.

The prevalence of positive LBT was significantly higher in the CP patients group compared with the control group (38.7 versus 2.5%; *P* < 0.01). A comparison of the mean values for CP patients and controls is shown in Figure 3. Baseline hydrogen values were significantly higher for the CP patients compared with controls (9.2 ± 8.9 versus 2.8 ± 2.2 ppm; *P* < 0.001). In fact, most of the time, mean hydrogen values were significantly higher in the CP group compared with the control group (Figure 3). Finally, the time lapse required to reach a colonic peak was also significantly longer in the CP patients compared with controls (131.1 ± 31.6 min versus 108.2 ± 20.6 min; *P* = 0.0043).

Tables 1 and 2 compare patients with a positive LBT with those with a negative LBT. The subgroups were comparable in terms of age, lifestyle, body mass index, etiology of CP, and symptom severity, including the M-ANNHEIM severity index. However, the two patients with the highest scores (M-ANNHEIM index = 16) had positive LBT. No statistically significant difference was found for the use of proton pump inhibitors (50.0% versus 52.6%; *P* = 0.99), narcotics (25.0% versus 21.1%; *P* = 0.99), and pancreatic enzyme replacement therapy (58.3% versus 47.4%; *P* = 0.72). Among the group of patients with CP, a trend toward an increased proportion of positive LBT in women was observed (66.6% versus 27.3%; *P* = 0.056).

## 4. Discussion

LBT was positive for 38.5% of patients with CP. To our knowledge, the present study was one of the largest to evaluate the prevalence of SIBO in CP in patients who did not undergo any previous gastric, pancreatic, or intestinal surgery. The prevalence of SIBO in our study was similar to that reported in previous studies, ranging between 22% and 67% [1, 13–15, 17]. One recent study investigating hydrogen and methane excretion measured by LBT reported a prevalence of 47.2% [26]. Two other recent studies using glucose breath test found a prevalence of 14.7% and 21% [27, 28].

To our knowledge, the present study was one of the few to report the prevalence of a methanogenic flora in a population with CP (present in 6.5% of patients). A recent study, using less stringent positivity criteria, also reported a prevalence of 5.9% of positive LBT only with methane and 29.4% of positive tests with both methane and hydrogen. Some tendency for hard stools and straining was observed in those patients [26]. Other studies reported that the production of methane was associated with slow intestinal transit and could present clinically with constipation [29–31]. Fifty percent of our patients who produced methane had also noted constipation.

The positivity thresholds of LBT and their sensitivity and specificity are highly inconsistent, ranging from 17% to 68% and 44% to 86%, respectively [32]. LBT is based on the assumption that standard intestinal transit time is approximatively 90 min and that a peak of the concentration of hydrogen or methane would indicate that the lactulose has reached the colonic bacteria. However, even with strict guidance regarding diet and avoidance of some medications, test results can be influenced by several factors, notably slow transit (with no peak at all over a 3 h period) or fast transit, implying that an early peak would represent a “colonic peak” and not SIBO. Moreover, up to 27% of healthy subjects do not show any peak during the test [33, 34]. In our cohort, nine (29.0%) CP patients and 12 (30.0%) controls did not show any peak in hydrogen excretion. However, among these CP patients, two showed an increased baseline hydrogen value and one showed increased methane production.

Nevertheless, an increase in the hydrogen or methane concentration of 20 ppm in the first 90 min is used in many studies and is a validated criterion [35, 36]. To increase the specificity of the LBT, we chose to increase the threshold to two consecutive increases of 13 ppm, which would correspond approximatively to a specificity of 86% according to a recent study [37].

Our results are similar to those from Kim et al. [26] and others [27, 28], demonstrating higher rates of positive LBT tests compared with other studies using the glucose breath test. Although the glucose breath test is potentially more specific than LBT (up to 83%) [23], because of the rapid absorption of glucose in the jejunum, the LBT has also been hypothesized to be more sensitive for ileal bacterial overgrowth, which cannot be correlated with the results of jejunal aspiration (gold standard) [38]. Despite all the pitfalls of the LBT and the lack of consensus regarding the cut-off points when compared with jejunal aspiration, this test has the advantages of being noninvasive, inexpensive, and safe for the patient and enables immediate interpretation.

CP appears to be associated with slow intestinal transit due to the use of narcotics or the effects of maldigestion on antroduodenal motility [39, 40]. Maldigestion is likely to increase the release of peptide YY by the ileum and inhibits jejunal motility. This “ileal brake” is described in tropical sprue, celiac disease, and CP but appears to be reversible in the latter with pancreatic enzyme replacement therapy [39, 41–44]. The higher baseline hydrogen value and the delayed time for a colonic peak in our CP patients may corroborate this hypothesis of slower intestinal transit. Although a significant proportion of our CP patients were on pancreatic enzyme replacement therapy, we did not find a difference in the number of positive tests or in the delay before the first peak between subgroups with and without pancreatic enzyme replacement therapy. The same was true for subgroup analysis of patients with diarrhea. These observations may suggest a lack of impact of exocrine pancreatic insufficiency in our cohort, although fat malabsorption was not measured.

No patient characteristic was predictive of the presence of SIBO, except for a possible association with female sex. This can be explained by the small size of our groups. Both CP and SIBO have similar clinical presentations, which could account for the absence of significant difference in the prevalence and severity of digestive symptomatology between groups with and without positive LBT. However, there was a trend toward more severe symptoms in the positive LBT group; both participants with the highest M-ANNHEIM score were in the LBT-positive group. Kim et al. [26] reported additional significant symptoms in their LBT-positive group, which included hydrogen and methane testing.

One possible limitation of the present study was the disparity in sex and age between the case and control groups. Although the control group was significantly younger and had more women than the CP group, we do not believe older age (mean age 53.8 years) was an important confounding factor because the risk for SIBO appears to increase mostly after 75 years of age [3, 45–47]. Furthermore, there were no age differences between our groups with positive and negative LBT. Although the diagnosis of CP was made using several imaging modalities, each may lead to a probable or definite diagnosis of CP according to specific characteristics [48]. Finally, CP rarely presents alone and many of our patients had other potential causes of SIBO including excessive alcohol intake [49], diabetes [50], celiac disease [51], cystic fibrosis [52], and cirrhosis [53] (Table 1). This, however, remains representative of the real population of CP patients.

## 5. Conclusion

A significant proportion of LBTs were positive in our population with CP compared with the control group, which is suggestive of SIBO. The effects of the treatment of SIBO on pain and pancreatic insufficiency remain to be studied.

## Figures and Tables

**Table 1:** Characteristics of patients with chronic pancreatitis (CP) and comparison between groups with positive (+) and negative (−) lactulose breath test (LBT).

	CP (*n* = 31)	LBT+ (*n* = 12)	LBT− (*n* = 19)	*P* (LBT+ vs LBT−)
Female sex	29.0	50.0	16.8	0.056
Age, years, mean ± SD	53.8	55.1 ± 17.2	53.4 ± 12.1	0.68
Caucasian	80.7	75.0	84.2	0.38
Active smokers	32.3	33.3	31.6	0.99
Active alcohol use	35.0	33.3	36.8	0.99
Alcohol-related CP	41.9	50.0	36.8	0.71
Idiopathic CP	32.3	33.3	31.6	0.99
Celiac disease	3.2	8.3	0	—
BMI overweight or obese	48.4	58.2	42.0	0.47
Radiation therapy	6.0	8.3	5.3	0.99
Insulin-dependent diabetes	12.9	8.3	15.8	0.99
Cirrhosis (Child Pugh A)	16.1	25.0	10.5	0.35
Cholecystectomy	19.4	25.0	15.8	0.65
Hepatic steatosis	32.0	16.7	42.1	0.24

Data is presented as % unless otherwise indicated. BMI: body mass index; vs: versus.

**Table 2:** Clinical manifestations in patients with chronic pancreatitis (CP) and positive (+) and negative (−) lactulose breath test (LBT).

Clinical manifestation	CP (*n* = 31)	LBT+ (*n* = 12)	LBT− (*n* = 19)	*P* (LBT+ vs LBT−)
Abdominal cramping	67.7 (25.8)	83.3 (41.7)	57.9 (15.8)	0.24
Dyspepsia	61.3 (16.1)	75.0 (25.0)	52.6 (10.5)	0.27
GERD, %	48.4	58.3	42.1	0.47
Bloating	48.4 (12.9)	50.0 (25.0)	47.4 (5.3)	0.99
Constipation	44.8 (13.8)	45.5 (9.1)	44.4 (16.7)	0.21
Epigastric pain	41.9 (22.6)	50.0 (33.3)	36.8 (15.8)	0.71
Chest pain, %	38.7	50.0	31.6	0.45
Early satiety, %	38.7	41.7	36.8	0.99
Steatorrhea, %	35.5	41.7	31.6	0.71
Anorexia, %	35.5	41.7	31.6	0.71
Diarrhea, %	9.7	16.7	21.1	0.99

Data is presented as % (% severe symptoms) unless otherwise indicated. GERD: gastroesophageal reflux disease; vs: versus.
